# Endothelial nitric oxide synthase-derived nitric oxide in the regulation of metabolism

**DOI:** 10.12688/f1000research.19998.1

**Published:** 2020-10-01

**Authors:** Margarita Tenopoulou, Paschalis-Thomas Doulias

**Affiliations:** 1 Children's Hospital of Philadelphia Research Institute, 3517 Civic Center Boulevard, Philadelphia, Pennsylvania, 19104-4318, USA; 2 Laboratory of Biochemistry, Department of Chemistry, School of Sciences, University of Ioannina, Ioannina, 45110, Greece

**Keywords:** endothelial nitric oxide synthase, metabolism, nitric oxide, nitric oxide signaling, protein S-nitrosylation

## Abstract

Nitric oxide is an endogenously formed gas that acts as a signaling molecule in the human body. The signaling functions of nitric oxide are accomplished through two primer mechanisms: cGMP-mediated phosphorylation and the formation of S-nitrosocysteine on proteins. This review presents and discusses previous and more recent findings documenting that nitric oxide signaling regulates metabolic activity. These discussions primarily focus on endothelial nitric oxide synthase (eNOS) as the source of nitric oxide.

## Biological synthesis of nitric oxide

Nitric oxide (NO) is an endogenously formed gas that is synthesized in nearly all cell types, tissues, and organs in the human body. NO is synthesized by the three isoforms of NO synthases (NOS), namely neuronal (nNOS or NOS1), inducible (iNOS or NOS2), and endothelial (eNOS or NOS3) ^1– 13^. These isoforms display cell type- and tissue-specific expression patterns. nNOS is expressed in cells of the central and peripheral nervous system as well as in epithelial cells of various organs, in pancreatic islet cells, and in vascular smooth muscle ^14, 15^. iNOS expression is, for the most part, stimulus evoked, although constitutive expression of the enzyme has been reported in monocytes and macrophages. Initially, the enzyme had been identified in macrophages; however, it has been demonstrated that iNOS expression can be induced in any cell type when the appropriate stimulus has been identified ^14, 16, 17^. Finally, eNOS is primarily expressed in endothelial cells; however, the protein has been identified in cardiomyocytes, platelets, human placenta, and kidney epithelial cells ^14, 16, 18^. All three isoforms utilize L-arginine as substrate and molecular oxygen and reduced nicotinamide-adenine-dinucleotide phosphate (NADPH) as co-substrates. All three isoforms utilize flavin adenine dinucleotide (FAD), flavin mononucleotide (FMN), and (6R)5,6,7,8-tetrahydro-L-biopterin (BH4) as cofactors. In the first step, L-arginine is hydroxylated followed by the oxidation of the hydroxylated intermediate, leading to the generation of NO and L-citrulline ^18– 20^. Multiple factors regulate the enzymatic activity of NOS, including substrate and cofactor bioavailability, calcium levels, protein levels, and dimerization as well as post-translational modifications. The molecular regulation of NOS is beyond the scope of this work, so readers are referred to excellent reviews ^18, 21– 29^. The concept of a mitochondrial NOS (mtNOS) emerged in the 90s. Initial reports ascribed mtNOS as a variant of eNOS followed by claims about a variant of iNOS, nNOS, or a completely unrelated enzyme. However, subsequent studies failed to document the existence of mtNOS. Nitrite (NO _2_^–^) represents a potential source of NO in the mitochondria, although the main sources of NO generation through nitrite are the red blood cells and the blood vessels ^30– 32^. Heme-containing enzymes can adopt a nitrite reductase activity to generate NO ^.^ under hypoxic conditions. These concepts are reviewed in 33.

## Molecular mechanisms facilitating the biological action of NO

In chemical terms, NO is a free radical, having an unpaired electron on the nitrogen atom. In contrast with other free radicals that are generated in biological systems, NO has relatively low chemical reactivity towards biomolecules and thus its half-life is longer ^34, 35^. This property facilitates the biological versatility and selectivity of NO. NO reacts with a number of enzymes and proteins. The most important physiological functions of NO are the activation of the soluble guanylate cyclase (sGC) and the post-translational modification of cysteine residues, a process known as S-nitrosation. Hydrogen sulfide (H _2_S) has emerged as an additional co-player of NO signaling. Evidence suggests that H _2_S and its metabolites influence NO formation and signaling through mechanisms that involve the regulation of eNOS expression and activity, the reaction with cyclic GMP (cGMP), and the activation of downstream kinases such as PGK-Ia. For recent advances in the field of H _2_S biology and its coordinated action with NO, readers are referred to 36, 37.

In the next two paragraphs, we discuss briefly cGMP and protein S-nitrosation formation as mediators of NO signaling. Subsequently, we focus on the regulation of metabolism via eNOS-derived NO signaling and protein S-nitrosation.

## cGMP-mediated signaling

The Nobel Prize in Physiology and Medicine in 1998 was awarded jointly to Drs Robert F. Furchgott, Louis J. Ignarro, and Ferid Murad for the discovery of NO in the cardiovascular system ^38^. Their studies revealed the molecular pathway by which endothelium-derived NO stimulates sGC in smooth muscle cells and the synthesis of cGMP. The intracellular elevation of cGMP levels is followed by a cascade of phosphorylation events leading to the dilation of blood vessels and the regulation of blood flow.

This signaling pathway, which is known as the “canonical signaling pathway of NO”, is regulated at several steps. NO is enzymatically synthesized and phosphodiesterases and phosphatases accomplish cGMP degradation and protein de-phosphorylation, respectively. In addition, another layer of regulation may exist at the level of NO transport from the endothelial to smooth muscle cells. Originally, it was proposed that upon its formation NO diffuses across the cellular membranes, reaching the smooth muscle cells ^39^. However, more recent studies propose a different model which initially requires the biotransformation of NO to S-nitrosothiol followed by the transport to smooth muscle cells and the activation of sGC ( 40 and references therein). Indeed, S-nitrosothiols have longer half-lives than NO and are strong activators of sGC ^41^.

Because of the central role of cGMP in the vasodilatory effects of NO, several drugs have been designed to enhance or attenuate cGMP-mediated signaling. Nitroglycerine and organic nitrates, a class of compounds that induce vasodilation, have been extensively used for the treatment of coronary artery disease ^42, 43^. On the other hand, inhibitors of phosphodiesterase 5 are successfully used for the treatment of erectile dysfunction ^44^.

## Signaling through protein S-nitrosylation

The addition or transfer of a NO equivalent to a reduced thiol is chemically defined as S-nitrosation. On the other hand, the existing nomenclature in the field of post-translational modifications uses the suffix “-ylation” to describe the biological function of these modifications. Conventionally, we opt to use the term S-nitrosylation throughout in order to be consistent with the existing nomenclature. The biological chemistries leading to S-nitrosylation *in vivo* remain unclear. Oxidation of cysteine thiol, metal-catalyzed transfer of NO, and exchange with small molecular weight (SMW) thiols represent three of the mechanisms that have been proposed to mediate protein S-nitrosylation in biological systems ^45, 46^. S-nitrosoglutathione (GSNO) is an endogenously formed S-nitrosothiol. The biological significance of GSNO as a mediator of protein S-nitrosylation *in vivo* is highlighted by the existence of GSNO reductase (GSNOR), an enzyme that regulates the intracellular levels of GSNO and indirectly protein S-nitrosylation ^47, 48^. In addition, protein-assisted transnitrosation has been proposed as a mechanism for S-nitrosylation *in vivo*. This mechanism has been elegantly described for the S-nitrosylation of caspase-3 by S-nitrosothioredoxin (Trx-SNO) ^49, 50^. In addition, thioredoxin has been reported to mediate the removal of nitroso group from proteins, a process called de-nitrosylation. Protein-mediated transnitrosation and de-nitrosation imply regulated processes which, for the most part, involve specific structural elements on the NO-donor and NO-acceptor proteins ^51^.

Comprehensive reviews on the chemistry of S‐nitrosocysteine formation have been recently published ^46, 52, 53^. It is very well documented that endogenous protein S-nitrosylation regulates protein function and biological responses ^54– 59^. However, a recent study argues against the notion that stable S-nitrosylation directly regulates protein function ^60^. The identification of the mammalian S-nitroso-CoA reductase system as a transducer of eNOS activity in reprogramming intermediary metabolism expands the functional components of NO signaling and represents a novel mechanism by which protein S-nitrosylation may regulate metabolic activity ^61^.

## eNOS-derived NO and metabolic activity

It is well recognized that eNOS-derived NO serves important biological functions, including the regulation of blood flow and vascular tone, the suppression of proliferation and migration of smooth muscle cells, and the interaction of leukocytes with endothelium ^18^. In addition to these functions, recent evidence indicates that eNOS-derived NO regulates metabolic activity ^62– 64^.

Endothelial dysfunction is a common feature in humans with cardiovascular risk factors and metabolic syndrome. Clinical studies in humans have shown that polymorphisms on the eNOS gene predispose for hypertension, insulin resistance, and type 2 diabetes, three of the major clinical features of metabolic syndrome ^65– 69^. In addition, studies in obese humans and animal models suggest that NO availability is decreased in adipose tissue and skeletal muscle owing to the diminished expression of eNOS protein ^70– 74^. The NO-generating capacity of eNOS is impacted by nutrient excess through mechanisms that involve the upregulated expression of negative regulators of eNOS activity such as caveolin-1 ^75, 76^. Moreover, studies in mice have shown that nutrient excess and obesity negatively regulate eNOS activity through the disruption of Akt-mediated signaling and thus the diminished phosphorylation of eNOS ^77– 80^. eNOS is a phosphoprotein. Serine 1177 in human sequence (S1176 in mouse sequence and S1179 in bovine sequence) represents the most abundant phosphoserine site ^80^. The protein kinase Akt phosphorylates eNOS at serine 1177 *in vivo*, whereas other kinases such as members of the family AGC (protein kinase A, protein kinase G, protein kinase C) as well as AMP-activated kinase (AMPK) can phosphorylate eNOS at the same site *in vitro* and perhaps *in vivo* ^81^. A proposed mechanism for the activation of eNOS through Akt involves the phosphorylation of Akt by the phosphatidylinositol 3-kinase (PI3K) ^82, 83^.

The aforementioned studies ^65– 74^ provide evidence that eNOS-derived NO acts as a signal that regulates metabolic activity. However, they do not provide mechanistic details in terms of which are the downstream effectors of NO signaling and which metabolic pathways are impacted. In this regard, studies in animal models with reduced or even the lack of endothelium-derived NO could be highly informative. Moreover, these models could serve as disease-mimicking models to test therapeutic approaches that will replenish the bioactive NO and will restore NO-mediated signaling.

Mice lacking eNOS have been generated and characterized. They are hypertensive, hyperlipidemic, insulin resistant, and display age-dependent increase of adiposity and body weight ^84– 87^. They also display lower energy expenditure and oxygen consumption as compared to wild-type counterparts ^70, 88^. eNOS null (eNOS ^–/–^) mice fed a high-fat diet demonstrate exacerbated non-alcoholic fatty liver disease (NAFLD) pathogenesis as compared to wild-type mice ^89, 90^. Moreover, the genetic deletion of eNOS in diabetic mice (db/db) worsens renal diabetes pathology, indicating that the impact of the absence of eNOS in the setting of diabetes is not limited to the aorta but also extends to the renal vasculature ^89, 91^. Genetic deletion of eNOS in mice induces anatomical alterations and impacts the metabolic activity and energetic profile of oxidative skeletal muscle. The soleus muscle displays decreased respiratory capacity in eNOS ^–/–^ as compared to wild-type mice ^91^. Studies by Kashiwagi *et al*. implicate AMPK–eNOS phosphorylation-activated formation of NO as a signal that impacts metabolic activity ^92^. Mice with an S1176A mutation on eNOS are unable to increase the biosynthesis of NO via AMPK-dependent phosphorylation. These mice develop insulin resistance and hypolipidemia and display increased body weight upon high-fat diet. On the contrary, mice harboring the S1176D mutation on eNOS, which mimics the effect of phosphorylation and thus results in a constitutively active enzyme, have normal levels of insulin and do not gain weight when they are fed a high-fat diet ^92^. Also, it has been shown that overexpression of the eNOS gene diminishes the sensitivity to diet-induced obesity and hyperinsulinemia via metabolic changes that occur at the adipose tissue ^93^. Collectively, mice with "diminished capacity to generate eNOS-derived NO" exhibit systemic and organ-specific metabolic derangements, including several features of metabolic syndrome. Therefore, studies in these mice are directly relevant to human disease and facilitate mechanistic insights and therapeutic interventions. At this point, we want to provide more information regarding the source of eNOS ^–/–^ mice that have been used in the aforementioned studies. Four different groups have generated mice with targeted deletion of the eNOS gene. The most consistent phenotype among these strains is hypertension. In the majority of the studies reported here, mice purchased from Jackson Laboratories were used ^85– 91, 94^. Studies by Huang’s group were performed in mice generated at Harvard University ^92, 94^.

The question that remains open is this: what are the mechanism(s) by which NO impacts metabolic activity? Published studies from others and our laboratory have provided insights into these mechanisms. Using mass spectrometry-based approaches, we precisely mapped the endogenous S-nitrosoproteomes in several organs of wild-type and eNOS ^–/–^ mice. Importantly, a clear dependency of endogenous S-nitrosylation from eNOS-derived NO was documented in all organs ^54^. Functional interrogation revealed clustering of S-nitrosylated proteins in several anabolic and catabolic processes, implicating cysteine S-nitrosylation as a global regulator of energy homeostasis. Of particular interest was the finding that nearly all the enzymes and transporters participating in the fatty acid oxidation pathway were targeted by S-nitrosylation in an eNOS-derived NO manner ^54^.

Fatty acid oxidation is the primary metabolic process for the generation of ATP in the heart, in the skeletal muscle during exercise, and in the liver under conditions of glucose scarcity ^95^. Our published work as well as work by others document that eNOS ^–/–^ mice have a reduced capacity to oxidize long-chain fatty acids in the heart, liver, and skeletal muscle ^54, 91, 94^. These observations prompt us to investigate the impact of NO signaling on the metabolic adaptations that occur during fasting. Our data document that young eNOS ^–/–^ mice have a normal response to fasting despite their inability to increase the fatty acid oxidation rate in the liver as compared to wild-type mice ^94^. Aged eNOS ^–/–^ mice exhibited metabolic derangements resulting in reduced utilization of fat to generate energy, lower resting metabolic activity, and diminished physical activity. These data suggest that eNOS–NO signaling is not essential for the metabolic adaptation to fasting; however, it is critical for regulating systemic metabolic homeostasis in aging ^94^.

In an attempt to understand better how NO signaling impacts protein function, we focused on very long-chain acyl-CoA dehydrogenase (VLCAD), the first enzyme of the long-chain fatty acid oxidation pathway. Data in mouse cells and tissue homogenates document the reversible S-nitrosylation of VLCAD on a single cysteine residue ^54, 96^. Kinetic studies document a 29-fold increase of catalytic efficiency of S-nitrosylated VLCAD as compared to the unmodified enzyme ^54^. Placing the kinetic findings into a biological context, we can infer that almost exclusively the S-nitrosylated molecules execute the dehydrogenation of long-chain fatty acids *in vivo* with very minimal, if any, contribution from the unmodified molecules. Recent data document that metabolic enzymes are post-translationally modified and the levels of modifications dynamically change in response to metabolic demands ^54, 97– 106^. Despite these sound data, their biological implications remain unclear. Specific questions regarding the impact of each modification on protein function, the prioritization of one modification over the other(s), and the impact on metabolic and energy homeostasis, organ specifically and systemically, are still open and further investigation is required.

The replenishment of bioactive NO has been used as a strategy to restore NO signaling and biological function. Currently, several clinical trials are testing the efficacy of bioactive NO (nitrite or nitrate) to improve cardiovascular function, improve physiological function in the elderly, and restore metabolic activity (NCT01681810, NCT02393742). Carlstrom and co-workers have shown that chronic nitrate treatment reduced visceral fat accumulation and circulating levels of triglycerides and improved glucose tolerance in eNOS ^–/–^ mice ^107– 109^. Recently, Lai *et al*. reported a beneficial effect of nitrite treatment in a rodent model of pulmonary hypertension associated with heart failure with preserved ejection fraction (HFpEF). The authors proposed that nitrite activates AMPK signaling and sirtuin 3 (SIRT3) through a mechanism that is independent from the formation of NO ^109^. Furthermore, studies from our group document that the chronic replenishment of bioavailable NO prevented age-dependent biochemical, metabolic, and phenotypic decline in eNOS ^–/–^ mice, indicating the critical influence of eNOS–NO signaling in maintaining metabolic homeostasis ^110^.

Collectively, preclinical findings in animal models support the notion that long-term replenishment of NO may be a suitable approach to correct metabolic diseases such as hypertension and metabolic syndrome.

## Future perspectives

This review focuses on the regulation of metabolic activity by NO signaling. In most responses that are indispensable for life, biological redundancy or co-regulation secures safe transitions that maintain biological functions. Thus, it is logical to infer that NO signaling via protein S-nitrosylation and other post-translational modifications is fully integrated in the metabolic cellular responses to allow for coordinated regulation of metabolism. Therefore, the investigation of this coordinated regulation represents an exciting research area. Delineating the physiological mechanisms that control metabolic activity and energy homeostasis will allow us to identify potential therapeutic targets in an attempt to improve the quality of life of individuals with metabolic disorders.

## Abbreviations

AMPK, AMP-activated kinase: cGMP, cyclic GMP; eNOS, endothelial nitric oxide synthase; eNOS ^–/–^, eNOS null; GSNO, glutathione S-nitrosothiol; H _2_S, hydrogen sulfide; iNOS, inducible nitric oxide synthase; mtNOS, mitochondrial nitric oxide synthase; NO, nitric oxide; NOS, nitric oxide synthase; nNOS, neuronal nitric oxide synthase; sGC, soluble guanylate cyclase; VLCAD, very long-chain acyl-CoA dehydrogenase.
